# Linkage Mapping Reveals QTL for Flowering Time-Related Traits under Multiple Abiotic Stress Conditions in Maize

**DOI:** 10.3390/ijms23158410

**Published:** 2022-07-29

**Authors:** Pengfei Leng, Siffat Ullah Khan, Dengfeng Zhang, Guyi Zhou, Xuhuan Zhang, Yanxiao Zheng, Tianyu Wang, Jun Zhao

**Affiliations:** 1 Biotechnology Research Institute, Chinese Academy of Agricultural Sciences, Beijing 100081, China; sifat.phd@gmail.com (S.U.K.); zhouguyi0805@163.com (G.Z.); zhangxuhuan01@163.com (X.Z.); zyx080507@163.com (Y.Z.); 2 Institute of Crop Sciences, Chinese Academy of Agricultural Sciences, Beijing 100081, China; zhangdengfeng@caas.cn (D.Z.); wangtianyu@caas.cn (T.W.)

**Keywords:** maize, linkage mapping, flowering time, drought stress, high density planting

## Abstract

Variation in flowering plays a major role in maize photoperiod adaptation during long-term domestication. It is of high value to investigate the genetic basis of maize flowering under a wide range of environmental conditions in order to overcome photoperiod sensitivity or enhance stress tolerance. A recombinant inbred line (RIL) population derived from a cross between Huangzaosi and Mo17, composed of 121 lines and genotyped by 8329 specifically developed markers, was field evaluated in two consecutive years under two planting densities (67,500 and 120,000 plants ha^−1^) and two water treatments (normal irrigation and drought stress at the flowering stage). The days to silking (DTS), days to anthesis (DTA), and anthesis to silking interval (ASI) were all evaluated. Within the RIL population, DTS and DTA expanded as planting density and water deficit increased. For DTA, DTS, ASI, and ASI-delay, a total of 22, 17, 21, and 11 QTLs were identified, respectively. More than two significant QTLs were identified in each of the nine chromosomal intervals. Under diverse conditions and locations, six QTLs (quantitative trait locus) for DTS and DTA were discovered in Chr. 8: 118.13–125.31 Mb. Three chromosome regions, Chr. 3: 196.14–199.89 Mb, Chr. 8: 169.02–172.46 Mb, and Chr. 9: 128.12–137.26 Mb, all had QTLs for ASI-delay under normal and stress conditions, suggesting their possible roles in stress tolerance enhancement. These QTL hotspots will promote early-maturing or multiple abiotic stress-tolerant maize breeding, as well as shed light on the development of maize varieties with a broad range of adaptations.

## 1. Introduction

Maize is one of the most widely grown crops in the world, from 40° S to 58° N, from below sea level to the altitude of 3000 m, and from the arid and semi-arid regions of Russia to an average annual rainfall of 6000 mm in India [1,2,3]. An ever-increasing global population is the biggest challenge, in light of the rapidly fluctuating climate environments [4]. It was estimated that around a 60% upsurge in yield would be required to nourish a global population of about 10 billion [5]. During long-term domestication, many of the landraces and commercial hybrids exhibited architectural traits, such as flowering time, growth duration, and adaptation to a wide range of geographies [2]. Flowering time is a key breeding goal for maize, since it shows adaptability to a particular environment and agro-ecological systems. Flowering time, on the other hand, had a substantial positive link with stover quality characteristics, such as in vitro organic matter digestibility, and was negatively correlated with cell wall digestibility traits, such as neutral detergent fiber (NDF) and silage maize NDF digestibility [6]. Polymorphisms in the monolignol biosynthetic genes and colocalized QTLs (quantitative trait locus) also confirmed the correlation between forage maize stover quality and flowering time [7,8]. Therefore, understanding the genetic basis of the flowering time in maize might not only benefit the grain yield improvement, but also the elite forage-maize cultivars’ breeding.

Flowering is a critical stage in a plant’s shift from vegetative to reproductive growth, and it occurs when the light, temperature, and water conditions are favorable. The projected severe drought-stressed conditions, resulting from the withdrawal of the water supply before the blossoming stage, leads to a significant reduction in the maize output [9,10]. Due to dehydration stress, tissue expansion in silk is influenced more than cell division, bringing about small silk cell size [11], which indicates that the rate of silk extension is highly reliant on plant water potential. Pollen shedding and silk elongation are inhibited in dehydrated environments, resulting in inefficient fertilization and substantial corn yield loss [12]. Traditional grain yield genetic improvement has reached a bottleneck, and high-density planting has emerged as a viable option for increasing output per unit area. With increased planting density, there is more competition for resources among the individual plants, such as water, light, and nutrients [13,14]. In maize, increased planting density slowed the silking and pollen shedding, resulting in fewer spikelet primordia that could be converted into functional florets at flowering [15]. High density planting has a greater impact on female ear development, causing delayed silking and female ear distinction. A study on the impact of density planting-stress on flowering time in maize could provide a theoretical basis for improving the yield per unit area under dense planting conditions. A prolonged period between the anther protrusion and silk appearance [16] occurs when the silk extrusion is even more delayed. Extended anthesis to silking interval (ASI) causes asynchronous flowering under variable circumstances [11,17], which is associated with a 40–50% yield loss [18,19,20,21]. ASI is a good secondary characteristic for drought tolerance in maize since it indicates plant vulnerability to abiotic stress and has a significant correlation with grain production, especially under drought stress [21,22,23]. As a result, shortening the growth period and avoiding hot temperatures and other seasons during the flowering period are critical for increasing the maize production and stress tolerance.

Flowering time is a common quantitative feature that influences the photoperiod adaptation in crops. Traditional approaches, such as QTL mapping and genome-wide association studies (GWAS), have been used to determine the genetic basis of the quantitative trait variation. A considerable number of minor-effect QTLs have been cloned so far, due to the complex genetic structure of the maize flowering time both under normal and stress conditions [2,24,25,26,27,28,29,30,31,32,33]. Meta-analysis aids in the identification and detection of some of the common QTLs across studies, resulting in interesting candidates for maize flowering time [34,35,36,37]. Multi-population mapping studies have increasingly evolved, as molecular marker technology has matured and the cost of genotyping has decreased [2,21,35,38,39,40,41,42].

In the United States-Nested Association Mapping (US-NAM) population, 36 QTLs for DTA and 39 QTLs for days to silking (DTS) were discovered through a joint linkage analysis on maize flowering [2]. With a single QTL having a minor effect, these QTLs could explain 89% of the phenotypic variation. Two sets of NAM populations (US-NAM and Chinese-NAM) and a natural population, evaluated under well-water and water-stress treatments, were used to identify the SNPs that were associated with flowering time-related traits [43]. These SNPs clustered within and 5 Kb region upstream of the gene, according to further study. Due to the limited influence of a single QTL, only a few QTLs for maize flowering time were fine mapped and cloned, in contrast to the large numbers of QTLs for maize flowering time. In maize GWAS, multiple association panels were employed, the majority of which are colocalized with known QTL intervals and were environment specific [44,45,46,47]. *Vgt1* [29], *ZmCCT* [38], *ZmCCT9* [48], *ZCN8* [49], and *ZmMADS69* [50] were classically cloned maize flowering time genes that were found either by QTL mapping or GWAS [51]. Other candidate genes that were not described were *dlf1*, *si1*, *ZAG1*, *ZCN12*, and *Zmm19* [37,51]. Recently, 33 hotspots associated with maize flowering under stressful conditions were unveiled, using an association panel of 300 inbred lines that were tested under drought stress, heat stress, drought–heat stress, and conventional conditions [21], providing valuable resources for the stress-tolerant maize breeding through marker-assisted selection and/or genomic selection.

Most of the flowering time genetic studies in maize were conducted under a single stress scenario. In this study, an existing RIL population was evaluated under a variety of field circumstances, including water treatments and planting densities. The goals of this research were to: (1) identify QTLs controlling maize flowering time; (2) detect stable stress-related QTLs for maize flowering; and (3) obtain consistent QTLs for ASI under diverse abiotic stresses.

## 2. Results

### 2.1. Phenotypic Evaluation of Traits Related to Flowering Time

The DTA, DTS, and ASI of the parental lines were recorded under a variety of environments, with variable water treatments (WW and WS) and planting densities (ND and HD) (Table 1). Similar performances for DTA exist in Huangzaosi and Mo17 under all of the growth conditions, with 75 days in Urumqi (Xinjiang province, 43°57′ N, 87°49′ E) and 67 days in Langfang (Hebei province, 39°35′ N, 116°35′ E), respectively. Mo17 had a higher DTS and ASI than Huangzaosi, especially under stress circumstances, indicating that its female silk extrusion was more susceptible to environmental stimulus than male blossoming. For Huangzaosi and Mo17, the drought stress slowed female silk extrusion by 1.7 and 5.1 days, respectively, whereas they were slowed by 0.9 and 4.3 days by the high planting density. Therefore, Huangzaosi, with a shorter flowering date and less ASI enlargement, is an abiotic stress-tolerant line compared to Mo17.

Under each type of management, the range of phenotypic distribution was large for the three traits examined, suggesting the broader diversity within the population (Table 1). The descriptive statistics, heritability estimates, and coefficient of variance for the phenotypic traits are listed in Table 1. ANOVA showed that a significant genotypic variation (*σ*^2^*_G_*) existed for the primary traits, including DTS and DTA, and also secondary traits ASI under all of the environments (Table 1). The genotype by environment variation (*σ*^2^*_GE_*) was also significant (*p* < 0.01), but not as pronounced as the variation among lines. All of the tested traits had increased *H^2^* (broad-sense heritability) across all of the environments, ranging from 64.33% (ASI-HD) to 86.94% (DTA-WW). The *H^2^* of the primary flowering time traits was higher than that of the secondary traits, ranging from 79.34% to 86.4% for the flowering date and 64.33% to 80.58% for ASI, indicating that the stably inherited genetic factors were critical during the establishment of these traits (Table 1). Meanwhile, the RIL population had approximately 10 days of flowering period, which was longer in Xinjiang than in Langfang, implying that the ecological environment differed substantially. The phenotypic characteristics of the RIL population were tested for normality under various water deficit and planting density conditions. The normality test revealed that the frequency distributions for the traits in the RIL population were nearly normal, as evidenced by the kurtosis and skewness factors.

**Figure 1:**
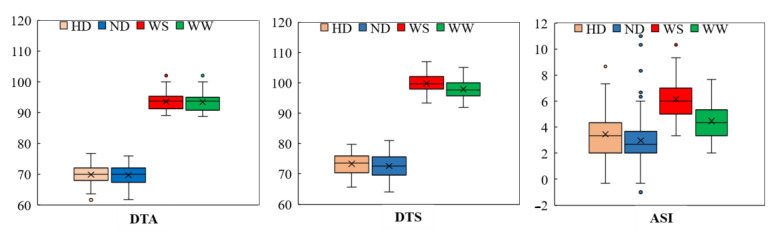
The boxplots of the phenotypic distribution of all of the 12-target trait-environment combinations between the three target traits, i.e., DTA (day); DTS (day); ASI (day) and the four evaluation conditions including well-water (WW); water-stress (WS); normal density (ND); and high density (HD).

The mean DTA value evaluated under the WW and ND conditions was comparable to that observed under the stress conditions (Table 1). The average DTA of the RIL population was 69.7 days under the ND conditions and 69.8 under the HD conditions (Table 1), indicating an almost negligible prolongation of anthesis date by high density planting. Under the two planting densities, the heritability of DTA was 84.25% and 80.68%, respectively (Table 1). Under the WW regime, the average DTA was 93.4 days across the two years, expanding to 93.6 days under the WS regime, with *H^2^* higher than 83.8%. In terms of the DTS, the mean value under the WS and HD was at least one day later than those under normal growth conditions. Under the WS regime, the average DTS in the RIL population was longer than under the WW regime, ranged from 99.8 days with a CV of 2.9% to 97.9 days with a CV of 3.0% (Table 1). With an estimated heritability of 83.12% and 79.34%, high density planting had a minor effect on the DTS, causing a one-day delay as compared to the ND condition. Nevertheless, the mean value of the ASI evaluated under the WW and ND conditions was lower than that evaluated under the stress conditions. Under both kinds of water treatment, the RIL population’s ASI is near to the normal distribution, but the distribution centers are different (Figure 1), indicating that the maize flowering, particularly for silk extrusion, was delayed under drought conditions. The average ASI in the RIL population was higher under the WS regime than under the WW regime (Figure 1). Under drought stress, the mean ASI values ranged from 3.3 to 10.3 days, with a CV of 26.5% (Table 1). The average ASI across the two years of the WS regime was 6.2 days, increasing significantly by 35.6% compared to the WW regime. The estimated heritability of ASI was greater than 76% under the two water treatments (Table 1), but it did not alter much under density planting conditions, which were 3.0 days under the ND and 3.5 days under the HD. Variations in traits were discovered, which might be due to fluctuating field environmental conditions or genotypic variations (Figure 1).

### 2.2. QTLs for Flowering Time-Related Traits in the RIL Population under Multiple Environments

**Figure 2:**
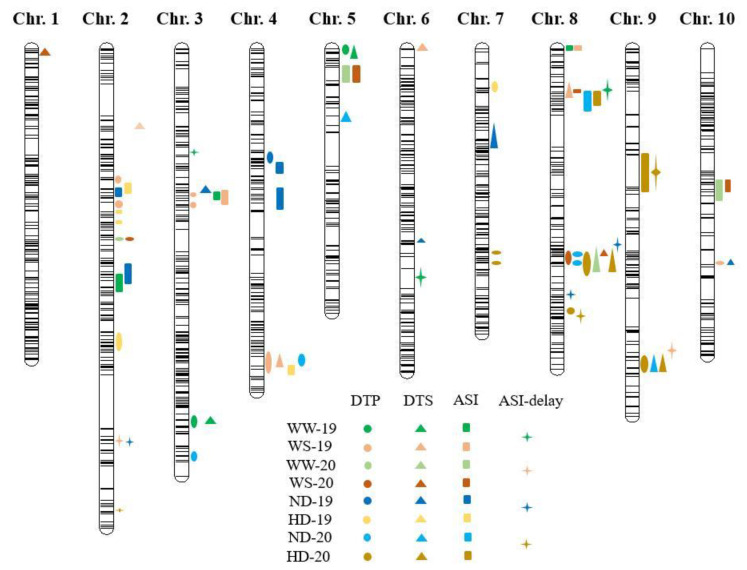
QTL for days to anthesis (DTA); days to silking (DTS); anthesis-silking interval (ASI); and ASI-delay evaluated under well-water (WW); water-stress (WS); normal density (ND); and high density (HD). Circles, triangles, squares, and masks represent the DTA, DTS, ASI, and ASI-Delay, respectively.

A total of 71 QTLs for the maize flowering-related traits were identified across the multiple environments. The DTA, DTS, ASI, and ASI-delay each had 22, 17, 21, and 11 QTLs, with LOD thresholds varying from 2.50–6.57 (Figure 2). For the DTA-WS-19 and the ASI-ND-19, the phenotypic variance explained by each QTL ranged from 8.34% to 33.68%.

#### 2.2.1. DTA

In total, 22 QTLs for the DTA were identified, distributed across 10 chromosomes, except for chromosomes 1 and 6, and accounting for 8.4% (DTA-WS-19) to 23.9% (DTA-HD-19) of the phenotypic variation (Table 2). These QTLs ranged in size from 0.24 Mb to 73.59 Mb on individual chromosomes (Table 2). Nine QTLs with PVE (phenotypic variation explained) > 10% were identified for the DTA under the WW and ND conditions. However, there was no common QTL for the four environments. Under the HD conditions, seven QTLs were found for the DTA, two for the DTA-HD-19, and five for the DTA-HD-20. Seven QTLs for the DTA, five for the DTA-WS-19, and two for the DTA-WS-20 were detected under WS conditions. The three stable QTLs, *qDTA2-3*, *qDTA4-1*, and *qDTA8-1*, were found in at least two environments. More than two water treatment-specific QTLs, *qDTA2-3* (Chr. 2: 62.10–69.71 Mb, Xinjiang), showed positive additive effects and were discovered under at least two water treatments, contributing 14.4% and 15.3% of the phenotypic variation in the WS and WW, respectively. The QTLs’ additive effects ranged from 2.47 to 3.33, indicating that it carries an allele that promotes pollen shedding and is inherited from the parent Huangzaosi. In the DTA-ND-20 and DTA-WS-19, *qDTA4-1* (Chr. 4: 21.69–22.98 Mb) was detected, with PVE ranged from 9.5–15.0%. The *qDTA8-1* (Chr. 8: 123.81–124.65 Mb) was identified to be expressed in three varied environments: DTA-ND-20, DTA-HD-20, and DTA-WS-20. With increased plant density and water deficit, the PVE (10.3–18.3%) and additive effect (1.03–2.14) of *qDTA8-1* enlarged, implying a favorable role during female flowering under abiotic stress conditions.

#### 2.2.2. DTS

A total of 19 QTLs influencing the DTS, distributed on all 10 chromosomes, were identified by single-environment mapping (Figure 2; Table 3). The individual QTL contributions to phenotypic variance ranged from 9.0% (*qDTS1*) to 24.2% (*qDTS5-1*) (Table 3). The physical distance of these QTLs on individual chromosomes ranged from 0.77–20.66 Mb in size. The positive additive effects ranged from 0.80 to 2.14 for five QTLs, whereas the negative additive effects ranged from −0.60 to 1.36 for thirteen QTLs. There were nine QTLs found with PVE > 10% for the DTS in normal conditions, and no common QTL for the four environments. In 2020, only two QTLs for the DTS on Chromosomes 8 and 9 were discovered in HD circumstances, explaining 12.8–25.6% and 10.9–15.9% of the phenotypic variation, respectively. The negative additive effect of *qDTS9* was observed. Under the WS condition, six QTLs were found for the DTS, four for the DTS-WS-19, and two for the DTS-WS-20, with only *qDTS8-2* showing a positive additive effect. The two stable QTLs, *qDTS3-2* and *qDTS8-2*, were detected in at least two environments. For DTS-ND-19 and DTS-WS-19, a QTL *qDTS3*, Chr. 3: 37.16–50.10 Mb, was discovered, generating 10.5–16.8% of the phenotypic variation and negative additive effects (−1.24 to −1.68). The *qDTS3-2* reduces silk extrusion in maize by carrying genes derived from the abiotic stress-sensitive parent, Mo17. Again, the major QTL located on chromosome 8 (*qDTS8-2*, Chr.8: 118.13–124.64, 20.13 Mb interval) was repeatedly identified under three environments: DTS-HD-20; DTS-WW-20; and DTS-WS-20 (Table 3). The *qDTS8-2* was one of the four QTLs positively associated with maize female flowering, with positive additive effect of 0.98–2.08. Both of the PVE enlarged with the increasing plant density and water deficit, indicating that an allele from the tolerant parent line Huangzaosi can positively regulate female blooming under abiotic stresses.

#### 2.2.3. ASI

A total of 13 QTLs controlling ASI were identified across 8 environments, distributed over all of the chromosomes, and 10 of them were found in only one environment or plant density (Figure 2; Table 4). The physical distance of these QTLs was 0.78–29.79 Mb in size. The individual QTL contributions to phenotypic variance ranged from 8.8% (*qASI8-2*) to 43.2% (*qASI4-2*) (Table 4). Five of the QTLs were found for ASI under normal conditions, while one integrated major QTL (*qASI2-1*, Chr. 2: 133.15–143.39 Mb) was found for ASI under the ND and WW conditions. The *qASI2-1* explained 14.2–27.8% of the phenotypic variation in ASI and showed positive additive effects (0.80–1.71), indicating that this allele was derived from the tolerant parent Huangzaosi and exhibited shorter ASI under normal growth conditions in both Hebei and Xinjiang. Likewise, six QTLs were found on Chromosomes 2, 4, 8, and 9 for the ASI under the HD conditions, with explained phenotypic variance ranging from 2.7% to 34.0%. Two QTLs in chromosomes 2 (*qASI2-2*, Chr. 2: 26.98–30.10 Mb) and 8 (*qASI8-3*, Chr. 8: 170.21–171.40 Mb) were commonly identified in ASI-HD-19 and ASI-ND-19, with the PVE ranging from 2.75–17.87% and 10.24–17.43%, respectively. ASI-WS-19 and ASI-WW-19 shared two QTLs in chromosomes 3 (*qASI3*, Chr. 3: 198.39–199.62 Mb) and 8 (*qASI8-1*, Chr. 8: 174.44–175.59 Mb), as well as one QTL in chromosome 10 (*qASI10*, Chr. 10: 120.82–127.08 Mb) for ASI-WS-20 and ASI-WW-20. These three QTLs accounted for more than 10% of the PVE and had a negative additive effect.

#### 2.2.4. ASI-Delay

A total of 11 QTLs influencing the ASI-D, distributed on the chromosomes 2, 3, 6, 8, and 9, were identified by single-environment mapping (Figure 2; Table 3). The physical distance of these QTLs was 0.78–29.79 Mb. The individual QTL contributions to phenotypic variance varied from 11.2% (*qASI-Delay2-2*, Chr. 2: 234.26–234.80 Mb) to 31.5% (*qASI-Delay8-3*, Chr. 8: 99.28–101.83 Mb) of total phenotypic variance (Table 5). Most of the QTLs were location-specific. Six QTLs on chromosomes 2, 3, 6, 8, and 9 were discovered for the ASI delay caused by high planting density. On chromosomes 2, 8, and 9, five QTLs for ASI delay caused by water stress were discovered. Surprisingly, one major QTL (*qASI-Delay2-1*) on Chr. 2, with a 2.00 Mb interval, was found to be associated with two abiotic stress conditions, 19-WS and 20-HD (Table 5). This QTL explained 19.4–30.0% of the PVE caused by water stress, and 12.6–13.8% of the PVE caused by density planting, while *qASI-Delay2-1* showing a stronger influence on ASI under water stress conditions.

### 2.3. Clusters of Colocalized Flowering Time QTLs

Five chromosome regions were identified to contain QTLs for at least two flowering traits after all of the QTLs were mapped onto the maize physical map. Under well-water conditions in 2019, two QTLs for DTA (*qDTA3-1*) and DTS (*qDTS3-1*) were found in regions Chr. 3: 9.78–11.63 Mb and Chr. 5: 168.86–171.88 Mb (Table 2 and Table 3), which inherited favorable alleles from Mo17 and Huangzaosi, respectively, and explained 8.69–22.77% of the phenotypic variation. This region exhibited a higher effect on the silking dates under well-water conditions than the pollen shedding under normal planting conditions. Two QTLs for DTA (*qDTA10*) and DTS (*qDTS10*) were found in Chr. 10: 136.09–139.00 Mb, explaining 23.87–24.27% PVE and with a positive additive effect. Three QTLs for DTA-HD-20 (*qDTA9*), DTS-HD-20 (*qDTS9*), and DTS-ND-20 (*qDTS9*) were found in Chr. 9: 84.29–117.32 Mb, explaining 10.47–14.48% PVE and having a negative additive effect. A hotspot region on Chr. 8 with a 6.42 Mb interval was detected for six overlapped QTLs, including DTA-HD-20 (*qDTA8-1*), DTA-ND-20 (*qDTA8-1*), DTA-WS-20 (*qDTA8-1*), DTS-HD-20 (*qDTS8-2*), DTS-WS-20 (*qDTS8-2*), and DTS-WW-20 (*qDTS8-2*), explaining 10.31–17.47% phenotypic variation. With increased planting density and water deficit, the positive additive effect of *qDTA8* enlarged, indicating an allele from a tolerant parent line.

### 2.4. Clusters of Colocalized QTLs for Abiotic Stresses

Three QTLs were found in Chr. 2: 23.74–40.00 Mb, two for ASI-19 at different planting densities and one for DTA-WS-19. These three QTLs inherited alleles from the sensitive parent Mo17 that correlated to drought tolerance and explained 2.95% to 17.87% of phenotypic variation. Two QTLs for *qASI-Delay9-2* and *qASI9*, which explained 14.70–22.7% of PVE and exhibited a positive additive effect, were found in Chr. 9: 128.12–137.26 Mb (Table 4 and Table 5). This QTL was only associated with density planting stress, showing that this allele was originated from the tolerant parent Huangzaosi and had a shorter ASI; it was therefore harder to be influenced by the high-density planting stress in Hebei. One QTL hotspot on Chr. 8 with a 2.23 Mb interval (170.22–172.46 Mb) was detected for five overlapped QTLs, including four for ASI, ASI-HD-20 (*qASI8-3*), ASI-ND-20 (*qASI8-3*), ASI-WS-20 (*qASI8-2*), and ASI-Delay-19 (*qASI-Delay8-1*), and one for DTS-WW-20 (*qDTS8-1*). This QTL was responsible for 8.95–17.43% of the phenotypic variation. A positive additive effect of *qDTA8* existed in correlation with ASI under a single environment, while negatively correlated with the DTS and ASI-Delay (−0.84 to −1.16).

## 3. Discussion

The maize flowering time was significantly associated with regional adaptation, evidenced by a large portion of flowering time SNPs correlated with altitude and latitude, respectively [52]. It is of great significance to overcome the photoperiod sensitivity and improve flowering adaptability. Previous studies found that maize is particularly vulnerable to abiotic stresses during flowering. A suitable flowering time can help maize avoid drought and high temperatures during its later growth period and make reasonable use of light energy to ensure its normal growth and yield production [53,54]. Secondary traits, such as ASI, have a greater heritability and a significant genetic association with GY, making them a preferable target for GY selection [21,55,56,57]. In this study, the ASI showed significant delay by drought stress and were less sensitive to density planting, consistent with previous reports [19,21,40,41,58,59,60,61]. As a result, maize grain that yields improvement through ASI improvement would be promisingly beneficial, especially under stressful conditions.

Although there existed smaller phenotypic variations between the parental lines Huangzaosi and Mo17, the flowering time-related characteristics of the RIL population from all of the environments exhibited extensive heterogeneity (Table 1). Increased planting density was accompanied by delayed DTA, DTS, and ASI. The DTS and ASI were more sensitive to drought stress than density planting, which showed consistency with other studies [21,40,41]. In this study, we found that the flowering time of both of the parental inbred lines and RILs was quite different in the two ecological regions. At the population level, the average difference was around 24 days for DTA, 25 days for DTS, and 1.4 days for ASI. On the one hand, this might be due to the plant’s adaptation to the latitude variation between the two ecological regions; on the other hand, it might be attributed to the lines’ sensitivity to light and temperature. The more sensitive it is to light and temperature, the greater the change in the growth period. It was found that the maize that adapted to high altitudes is more likely to bloom a week earlier than the wildland-adapted maize [62]. On the other hand, it is possible that the large ambient temperature difference between the day and night in Xinjiang, and the longer daytime duration from June to September, accompanied by high temperatures and dry heat, might extend the plant growth period. Drought stress increased the DTS by two days on average, whereas high density planting had a smaller impact. However, the ASI was less sensitive to the increasing plant density compared to the water deficit, which caused an average 0.5- and 1.7-day delays (*p* = 7.8 × 10^−31^). One explanation might be the effects of the different stresses on plant growth and development could be evidenced by the number of QTLs identified for each condition. In all, six QTLs were discovered for the DTS in the WS conditions and only two QTLs under HD conditions, while the DTA and ASI contained about the same number of QTLs. Stress conditions yielded a higher number of QTLs than the normal growth conditions. This was in line with the findings of Ribaut et al. [26], who found more QTLs under stress conditions than under normal irrigation conditions.

Drought stress has been identified as the most significant worldwide environmental constraint to maize yields. The maize flowering time under higher planting densities is less reported and seems to be regulated by more complex pathways, rather than controlled by several major genes or through an independent pathway. This study corroborated many previously known genes or loci. Three QTLs were found in Chr. 2 (23.74–40.00 Mb), two for ASI-19-ND, -HD, and one for DTA-WS-19. *AC208663.3 FG002* (*GRMZM2G473757*) encoding a NAC-transcription factor fell in this interval and was associated with the flowering stage under water deficit, high temperature, and combined stress conditions [21]. The NAC family transcription factors NAC050 and NAC052 were shown to be associated with histone demethylase JMJ14 in Arabidopsis and involved in flowering time regulation [63]. Another reported gene *GRMZM2G021560* was located in this interval, which was involved in both the photoperiod and vernalization pathways controlling flowering time, as identified by Xu et al. [36].

Three QTLs for DTA-HD-20, DTS-HD-20, and DTS-ND-20 were found in the Chr. 9: 84.29–117.32 Mb interval, explaining the 10.47–14.48% PVE. The consensus regions for photoperiod sensitivity generated by meta-analysis, *CQTL21* and *CQTL22* [36], hosted the most homologous genes, and were co-localized in our QTL hotspot in Chr. 9. *TED4* [64], *CRY1* [65], and *COL16* [66], etc. One QTL hotspot, Chr. 3: 9.78–11.63 Mb, harbored two QTLs for DTA-WW-19 and DTS-WW-19. Consistent with this finding, Wang et al. identified a QTL cluster in Chr. 3, with a genetic interval from 7.15–11.85 Mb in bin 3.02–3.03 [40]. This cluster contains two QTLs for ASI and DTS. Six QTLs for DTA-HD-20, DTA-ND-20, DTA-WS-20, DTS-HD-20, DTS-WS-20, and DTS-WW-20 were located in one QTL hotspot Chr. 8: 118.13–124.65 Mb. A similar genetic region located in Chr. 8 (123.50–142.36 Mb) was shared between two planting densities of 16,500 and 49,000 plants ha^−1^ through both bulk segregant analysis sequencing, using phenotypically extreme individuals from the intermated B73 × Mo17 (IBM) Syn14 population, and linkage mapping, using 224 IBM recombinant inbred lines [67]. Wang et al. also found a QTL cluster in Chr. 8, with a genetic interval of 107.40–122.55 Mb in bin 8.03–8.04 [40]. This cluster contains two QTLs for DTA and DTS, as well as a previously discovered flowering time locus, *vgt2* [68,69]. Despite the high confidence intervals, this large QTL hotspot proved critical to maize flowering under diverse stress conditions and may also contribute to the maize photoperiod adaptation. Serval discovered QTLs for the flowering period, such as days to pollen, located in bin 8.03–8.04 [24]. In 2019, another region in Chr. 10: 136.09–139.00 Mb harbored two QTLs for DTA and DTS under normal density planting conditions. Wang et al. discovered a stable QTL cluster in bin 10.05–10.07 of Chr. 10, where four QTLs for DTT, DTS, DTA, and TAI were detected at different planning densities [40]. Although the favorable effect of this stable QTL increased with plant density, it was not identified in our investigation at high planting density, possibly due to the varied parental lines. In this region, *ZFL1* (*GRMZM2G098813*), a homolog of *FLORICAULA*/*LEAFY,* was found to be involved in the flower development through meta-analysis [36]. Serval reported QTLs for the flowering time in maize localized in bin 10.05–10.07, such as days to pollen [24].

Besides the consensus QTL for flowering time and ASI, we also detected chromosome regions containing genetic loci for ASI-delay caused by abiotic stress. Two QTLs for ASI-DELAY-20 (*qASI-Delay9-2*) and ASI-HD-20 (*qASI-Delay9-1*), as well as a previously cloned gene *ZCN8* modulating maize flowering [49], were identified in Chr. 9: 128.12–137.26 Mb, explaining 14.70–16.11% of PVE. Meta-analysis revealed the phytochrome gene *PHYB2* (*GRMZM2G092174*) in this region, which responds to changes in the ambient light conditions [36]. Serval reported QTLs for the flowering time in maize localized in bins 8.03–8.04, including days to pollen, ear length of 21, ear diameter of 10, grain weight of 29, and a known flowering-time gene, *vgt2* [24,68,69,70]. Under normal and stress conditions, three QTLs for ASI and ASI-Delay were found in two chromosome regions, Chr. 8: 169.02–172.46 Mb and Chr. 3: 196.14–199.89 Mb (Table 1). Recently, an uncharacterized protein and pectin-esterase were found to be associated with ASI-Delay-17 and ASI-Delay-20 through GWAS, respectively [42]. The ASI-delay was caused by drought stress under the same experimental design as in this study. Taken together, these QTL hotspots may be meaningful for maize flowering under a variety of abiotic stress conditions, and thus contribute to photoperiod adaptation in maize.

In addition to the known genes for flowering time, a substantial number of QTLs for plant height and yield-related traits fell in the flowering time QTL hotspots identified in this study, such as Chr. 5: 160.17–171.88 Mb and Chr. 9: 8.43–11.74 Mb. For instance, the QTLs for plant height, test weight, kernel row number, kernel length, ear length, ear diameter, and grain yield [71,72,73,74], are located in the two above-mentioned chromosome intervals. Those QTLs, associated with plant height and yield overlap with the flowering period, could be due to a close correlation between flowering time, plant height, and yield-related traits. Despite the large confidence intervals, these QTL hotspots may be important for maize flowering under diverse stress conditions and may also contribute to maize photoperiod adaptation. It is possible that there is pleiotropy in these intervals, considering the relationship between the flowering time and plant height and yield-related traits.

## 4. Materials and Methods

### 4.1. Plant Materials

For linkage mapping, a panel of 121 recombinant inbred lines (RILs) was used, which were produced from a cross between Huangzaosi and Mo17 by single-seed descent and continuous selfing to the F_7_ generation. The RIL population was provided by Dr. Tianyu Wang, the Institute of Crop Science (ICS), Chinese Academy of Agricultural Sciences (CAAS), China. The RILs were genotyped by 78,507 SNPs, getting a high-resolution linkage map with 1262 bin markers [43]. The genetic map was 1524.53 cM (centiMorgans) in length, with a mean distance of 1.21 cM between adjacent markers.

### 4.2. Field Trials and Phenotyping

The field trials with WW (well-water) and WS (water-stress) treatments were conducted in Urumqi (Xinjiang province, 43°57′ N, 87°49′ E), as described by Khan et al. [42]. The RIL population was tested in 2019 and 2020. Each plot was 3.6 m in length, with a 0.24 m planting space. The spacing between the rows was 1.1 m. Every line was planted in two adjacent rows, one for WS and the other for WW, each with its own irrigation valve. Managed stress management was described by Khan et al. [42]. Briefly, irrigation was withdrawn at −21 D (days before flowering), estimated according to the Growing Degree Days (GDD), and soil moisture was maintained at 150–200 centibars from −7 D to 14 days post flowering. In Langfang (Hebei province, 39°35′ N, 116°35′ E), the same population was examined in 2019 and 2020 at normal (67,500 plants ha^−1^, ND) and high (120,000 plants ha^−1^, HD) planting densities, as described by Zhang et al. [75]. All of the plants were planted in three independent repeats, each with 15 lines.

The DTA and DTS were determined by the number of days from planting to 50% plants shedding pollen and having clearly visible silks in each repeat. The plants were considered to have reached anthesis and silking when one anther extruded (termed as pollen shed) or one silk was visible, respectively. ASI was calculated as the interval between the pollen shedding date and the silking date (ASI = DTS–DTA). The ASI-delay caused by abiotic stress was calculated as ASI-WS–ASI-WW or ASI-HD–ASI-ND.

### 4.3. Linkage Mapping Analysis

The linkage map and anchoring of markers were completed by Dr. Chunhui Li, ICS, CAAS, China. The RIL population’s SNP calling, filtering, and imputation stages were described previously [43]. The bin interval below 5 Kb was combined with the adjacent intervals to obtain the preliminary bin map. The *R* software package (Vienna, Austria, https://www.r-project.org/ (accessed on 11 August 2021)) was used to fill the genotype to obtain the high accuracy bin map. The bin interval of the bin map was utilized as a new marker to construct the genetic linkage map of the RIL population. The linkage mapping for the RIL population was conducted by model 6 of the composite interval mapping (CIM) in WinQTL Cartographer V2.5 [76], with a high-density bin-reported genetic map [43]. In this model, the algorithm with 1 cM walking speed and a 10 cM window was applied. The LOD (logarithm of odds) threshold was determined with 1000 permutations and at a level of significance of 0.05 [77]. The threshold of LOD >2.5 was adopted for declaring a QTL for each flowering time trait. For the single environment QTL mapping analysis, the phenotypic data were composed of the means of three replications. In this study, different QTLs for different traits and overlapping confidence intervals for different abiotic stress treatments were considered to be a QTL hotspot or a pleiotropic QTL. In particular, a QTL identified for a single treatment over multiple years or for different traits was considered to be a stable QTL. A location-specific and stress-specific QTL was defined as a QTL identified in only one location or one treatment. All of the QTLs were mapped onto the maize B73 RefGen_v2 (www.maizegdb.org/ (accessed on 12 October 2021)).

### 4.4. Statistical Analysis

Using the *Q*-test in Excel, the phenotypic data were further filtered by deleting the suspicious values from each replicate [75]. The mean value of the three replicates in each environment was used for association analysis. The best linear unbiased estimators (BLUPs) were calculated, using the genotype and covariate as fixed factors, whereas the remainder were random factors. The correlation coefficients were obtained based on the BLUE using Pearson’s statistic, applying the *cor* function of *R* software. Analysis of variance (ANOVA), correlation analysis, and broad sense heritability (*H^2^*) were estimated, using SPSS version 22 [78], according to the following equation:

$$H^{2}=\frac{\sigma_{G}^{2}}{\sigma_{G}^{2}+\sigma_{GE}^{2}/n+\sigma_{e}^{2}/nr}$$

where *σ*^2^*_G_* represents genetic variation; *σ*^2^*_GE_* shows the genotype × environment variation; *σ*^2^*_e_* shows residual error variation; *n* shows the number of environments; and *r* shows number of replicates [79].

## 5. Conclusions

The variation in flowering time helped maize adapt to a wide range of geographic environments during its long-term domestication. Study of the genetic basis of maize flowering, as well as the mining of superior alleles, is critical for a better understanding of maize domestication and adaptability, as well as crop genetic improvement. In total, 11 QTL hotspots for flowering time-related traits were discovered across multiple environments, each containing more than two QTLs. Three chromosome regions, Chr. 3: 196.14–199.89 Mb, Chr. 8: 169.02–172.46 Mb, and Chr. 9: 128.12–137.26 Mb, harbored QTLs for ASI-delay caused by drought stress or high-density planting. Taken together, the significant findings of the genetic intervals in our research will be of great importance for mining the flowering-time gene in maize, breeding maize varieties with broad photoperiod adaptation, as well as abiotic stress resistance lines via marker-assisted breeding.

## Figures and Tables

**Table 1:** Descriptive statistics, estimates of variance components, and heritability estimates for the flowering time traits of RIL population and its parental lines.

Traits	Treatments	Huangzaosi	Mo17	RIL-Population								
				Mean ± SD	Range	Skewness	Kurtosis	CV (%)	*H*^2^ (%)	*σ* ^2^ *_G_*	*σ* ^2^ *_GE_*	*σ* ^2^ *_e_*
DTA	WW	74.3	74.7	93.4 ± 2.8	88.7–102.0	0.41	0.49	3.0	86.94	23.08 ***	5.68 ***	0.76
	WS	74.3	75.0	93.6 ± 2.8	89.0–102.0	0.35	0.49	3.0	83.82	23.32 ***	6.75 ***	0.75
	ND	66.3	67.0	69.7 ± 3.1	61.7–76.0	−0.18	−0.36	4.43	84.25	27.03 ***	4.10 ***	12.01
	HD	66.7	66.0	69.8 ± 2.9	61.7–76.7	0.06	−0.27	4.14	80.68	33.71 **	7.26 **	20.64
DTS	WW	76.3	81.3	97.9 ± 2.9	92.0–105.0	−0.21	0.20	3.0	84.61	25.55 ***	7.84 ***	1.35
	WS	77.7	86.7	99.8 ± 2.9	93.3–107	0.29	0.17	2.9	81.28	25.61 ***	9.36 ***	1.30
	ND	67.7	71.0	72.6 ± 3.6	64.0–81.0	−0.48	0.01	5.02	83.12	38.96 ***	8.67 ***	15.47
	HD	69.3	74.3	73.3 ± 3.3	65.7–79.7	−0.67	−0.08	4.57	79.34	50.04 ***	16.13 ***	23.8
ASI	WW	2	6.6	4.4 ± 1.4	2.0–7.0	−0.36	0.33	32.0	80.58	6.31 ***	1.83 ***	0.64
	WS	3.7	11.7	6.2 ± 1.6	3.3–10.3	−0.42	0.36	26.5	76.84	8.42 ***	3.90 ***	0.54
	ND	1.4	4.0	3.0 ± 2.2	−1.0–11.0	3.31	1.37	73.08	65.98	8.01 ***	6.43 ***	2.49
	HD	2.3	8.3	3.5 ± 2.0	−0.3–8.7	0.22	0.69	57.46	64.33	9.99 ***	8.28 ***	2.40

DTA, days to anthesis; DTS, days to silking; ASI, anthesis–silking interval; WW, well-water; WS, water-stress; ND, normal density; HD, high density; SD, standard deviation; CV (%), coefficient of variation; *H*^2^ (%), broad-sense heritability; *σ*^2^*_G_*, genetic variation; *σ*^2^*_GE_*, genotype × environment variation; *σ*^2^*_e_*, residual error variance; **, *** Significant at *p* < 0.01 and *p* < 0.001.

**Table 2:** QTLs identified for DTA under two water regimes and planting densities during 2019 and 2020.

Treatment	QTLs	Chr.	Interval (Mb)	LOD	*R*^2^ (%)	Add Range	Phy-Dis (Mb)
WW-19	*qDTA3-1*	3	9.78–11.63	2.98	8.40–9.51	−0.83 to −0.89	1.85
	*qDTA5*	5	171.88–168.86	3.44	13.0–15.7	1.01–1.11	3.02
WS-19	*qDTA2-1*	2	23.74–26.98	2.81	7.9–13.3	−1.46 to −1.14	3.24
	*qDTA3-2*	3	199.27–199.62	2.81	10.1–10.7	1.11–1.14	0.35
	*qDTA3-3*	3	189.53–196.14	3.34	9.7–12.4	1.04–1.20	6.61
	*qDTA4-1*	4	17.23–22.98	3.91	11.1–15.0	1.22–1.43	5.75
	*qDTA10*	10	137.49–139.00	3.01	10.4–11.1	1.13–1.17	1.51
ND-19	*qDTA4-2*	4	194.30–211.05	4.12	15.0–21.3	−2.53 to −1.66	16.74
HD-19	*qDTA2-2*	2	180.83–186.27	4.05	15.7–20.8	−1.98 to −1.65	5.43
	*qDTA7-1*	7	9.53–13.98	4.61	18.7–27.2	−2.05 to −1.72	4.45
WW-20	*qDTA2-3*	2	62.10–69.71	3.75	14.8–15.3	2.70–3.28	7.61
WS-20	*qDTA2-3*	2	62.10–69.71	3.25	12.0–14.4	2.47–3.33	7.61
	*qDTA8-1*	8	118.89–125.31	3.88	12.9–18.3	1.03–1.23	6.42
ND-20	*qDTA3-4*	3	4.68–6.74	3.28	10.1–13.3	−1.15 to −1.01	2.06
	*qDTA4-1*	4	21.69–22.98	2.65	9.5–9.8	1.04–1.07	1.29
	*qDTA8-1*	8	123.81–124.65	2.87	10.3–10.8	1.05–1.07	0.84
	*qDTA8-2*	8	118.89–119.37	3.46	10.4–12.8	1.04–1.16	0.48
HD-20	*qDTA7-2*	7	147.89–151.25	3.00	8.8–10.2	−0.98 to −0.90	3.36
	*qDTA7-3*	7	153.85–155.25	3.26	9.3–11.0	−1.04 to −0.95	1.40
	*qDTA8-1*	8	118.13–124.65	7.79	14.3–31.1	1.46–2.14	6.52
	*qDTA8-3*	8	52.08–71.29	2.73	8.6–9.2	−1.22 to −1.10	19.20
	*qDTA9*	9	8.43–11.74	3.19	9.0–11.0	−1.01 to −0.91	3.31

DTA, days to anthesis; WW, well-water; WS, water-stress; ND, normal density; HD, high density; Add, additive effect; Phy-dis, physical distance.

**Table 3:** QTLs identified for DTS under two water regimes and planting densities during 2019 and 2020.

Treatment	QTLs	Chr.	Interval (Mb)	LOD	*R*^2^ (%)	Add Range	Phy-Dis (Mb)
WW-19	*qDTS3-1*	3	9.78–11.63	6.05	19.4–25.0	−1.56 to −1.40	1.85
	*qDTS5-1*	5	167.01–171.88	6.84	13.9–24.2	1.20–1.56	4.87
WS-19	*qDTS2*	2	10.48–12.10	4.72	12.9–16.8	−1.60 to −1.44	1.63
	*qDTS3-2*	3	34.55–108.14	4.17	10.5–14.4	−1.46 to−1.24	73.59
	*qDTS6-1*	6	3.68–6.26	6.16	12.3–23.2	−1.78 to −1.32	2.58
	*qDTS8-1*	8	170.22–171.75	3.25	9.0–11.0	−1.25 to −1.14	1.53
ND-19	*qDTS3-2*	3	37.16–50.10	3.94	15.9–16.8	−1.68 to −1.66	12.94
	*qDTS6-2*	6	150.02–151.03	3.02	12.1–12.2	−1.46 to −1.47	1.01
	*qDTS7*	7	26.00–79.64	5.32	15.4–25.0	−2.12 to −1.75	53.65
	*qDTS10*	10	136.09–139.00	5.66	20.3–27.5	1.76–4.35	2.92
WW-20	*qDTS8-2*	8	118.13–126.48	4.73	11.1–17.5	0.98–1.27	8.35
WS-20	*qDTS1*	1	3.02–3.28	2.89	9.4–10.0	−0.98 to −0.95	0.26
	*qDTS8-2*	8	122.42–123.54	2.77	10.1–10.5	1.33–1.38	1.12
ND-20	*qDTS5-2*	5	65.94–81.3	3.15	11.2–12.2	1.25–1.32	15.10
	*qDTS9*	9	8.43–11.74	4.22	11.9–17.0	−1.49 to −1.26	3.31
HD-20	*qDTS8-2*	8	118.13–124.65	5.83	12.8–25.6	1.45–2.08	6.52
	*qDTS9*	9	8.43–11.73	3.87	10.9–15.9	−1.40 to −1.18	3.30

DTS, days to silking; WW, well-water; WS, water-stress; ND, normal density; HD, high density; Add, additive effect; Phy-dis, physical distance.

**Table 4:** QTLs identified for ASI under two water regimes and planting densities during 2019 and 2020.

Treatment	QTLs	Chr.	Interval (Mb)	LOD	*R*^2^ (%)	Add Range	Phy-Dis (Mb)
WW-19	*qASI2-1*	2	133.15–154.59	5.48	14.2–27.8	0.80–1.54	21.34
	*qASI3*	3	198.39–199.62	4.50	11.7–18.7	−1.36 to −0.94	1.23
	*qASI8-1*	8	174.37–175.59	2.76	10.1–11.0	−0.62 to −0.60	1.22
WS-19	*qASI3*	3	196.14–199.89	3.82	9.8–13.8	−1.09 to −0.90	3.74
	*qASI8-1*	8	174.40–175.59	3.33	10.7–11.9	−1.04 to −0.98	1.19
ND-19	*qASI2-2*	2	26.98–40.00	3.49	17.0–18.7	−1.35 to −1.29	13.02
	*qASI2-1*	2	113.08–143.39	5.80	21.2–36.1	1.33–1.71	30.31
	*qASI4-1*	4	188.52–191.77	3.89	16.4–18.7	−1.28 to −1.13	3.25
	*qASI4-2*	4	180.25–185.64	6.77	24.2–43.2	1.49–1.81	5.40
HD-19	*qASI2-2*	2	24.37–31.00	3.11	3.0–3.3	1.81–2.46	6.63
	*qASI2-3*	2	41.89–45.76	7.99	39.6–62.2	−7.65 to −5.133	3.87
	*qASI2-4*	2	50.82–54.36	3.03	30.2–34.0	−5.33 to −4.72	3.55
	*qASI4-3*	4	14.63–17.52	2.65	2.7–2.8	0.82–0.83	2.89
WW-20	*qASI5*	5	160.17–165.16	5.00	9.8–18.3	0.46–0.62	4.98
	*qASI10*	10	120.82–127.94	9.78	22.0–39.6	−0.96 to −0.70	7.11
WS-20	*qASI5*	5	160.17–165.16	4.78	11.6–18.0	0.59–0.72	4.98
	*qASI8-2*	8	171.40–171.65	2.70	8.8–9.3	0.53–0.55	0.24
	*qASI10*	10	120.82–127.08	3.62	9.9–13.6	−0.66 to −0.56	6.26
ND-20	*qASI8-3*	8	169.02–171.40	4.47	12.5–20.0	0.70–0.91	2.39
HD-20	*qASI8-3*	8	170.21–171.40	3.05	10.2–12.0	0.61–0.65	1.20
	*qASI9*	9	128.12–137.26	3.96	10.8–16.5	0.64–0.78	9.14

ASI, anthesis-silking interval; WW, well-water; WS, water-stress; ND, normal density; HD, high density; Add, additive effect; Phy-dis, physical distance.

**Table 5:** QTLs identified for ASI-delay caused by water stress and high planting density during 2019 and 2020.

Treatment	QTLs	Chr.	Interval (Mb)	LOD	*R*^2^ (%)	Add Range	Phy-Dis (Mb)
19-HD	*qASI-Delay3*	3	212.11–212.84	3.20	11.7–14.2	−0.94 to −0.86	0.72
	*qASI-Delay6*	6	157.38–158.59	2.78	12.0–13.0	−0.87 to −0.84	1.21
	*qASI-Delay8-1*	8	170.22–172.46	3.74	11.5–16.0	−1.02 to −0.83	2.23
20-HD	*qASI-Delay2-1*	2	210.00–216.61	3.07	12.6–13.8	−0.46 to−3.40	6.61
	*qASI-Delay9-1*	9	11.72–13.46	4.71	12.8–22.4	27.6–35.9	1.73
19-WS	*qASI-Delay2-1*	2	214.61–216.61	4.57	19.4–30.0	−3.18 to −2.50	2.00
	*qASI-Delay8-2*	8	124.65–140.01	4.57	19.7–31.1	2.48–3.58	15.36
	*qASI-Delay8-3*	8	99.28–101.83	4.75	21.7–31.5	−3.43 to −3.14	2.55
20-WS	*qASI-Delay2-2*	2	234.26–234.80	2.61	11.2	−0.43	0.54
	*qASI-Delay8-4*	8	25.01–52.08	3.45	11.3–14.7	−0.49 to −0.42	27.08
	*qASI-Delay9-2*	9	14.63–17.52	4.61	15.3–22.7	0.50–0.60	9.14

ASI-Delay, anthesis-silking interval delay; HD, high density; WS, water-stress; Add, additive effect; Phy-dis, physical distance.

## Data Availability

The data presented in this study are available on request from the corresponding author.
